# Prevalence and profile of diabetes kidney disease according to different diagnostic criteria in type 2 diabetes mellitus patients

**DOI:** 10.1186/1758-5996-7-S1-A28

**Published:** 2015-11-11

**Authors:** Sabrina Coelli, Ariana Aguiar Soares, Camila Kümmel Duarte, Ana Marina Moreira, Luiza Barboza de Souza, Andrea Carla Bauer, Themis Zelmanovitz, Sandra Pinho Silveiro

**Affiliations:** 1 Universidade Federal do Rio Grande do Sul (UFRGS), Porto Alegre, Brazil

## Background

Diabetes kidney disease (DKD) is the worldwide leading cause of end-stage renal disease. Diagnostic criteria have been recently revised.

## Objective

The aim of this study was to evaluate the prevalence and clinical profile of type 2 DM patients according to the employed definition of DKD: previous diagnostic criteria as compared to the present one.

## Materials and methods

566 type 2 DM outpatients from the Endocrine Unit ambulatory were included. DKD was defined by the presence of elevated urinary albumin excretion alone (UAE; >14 mg/l) -previous definition- or by the presence of elevated UAE and/or reduced (<60 ml/min/1.73 m2) glomerular filtration rate (GFR) -present definition.

## Results

**Figure 1:**
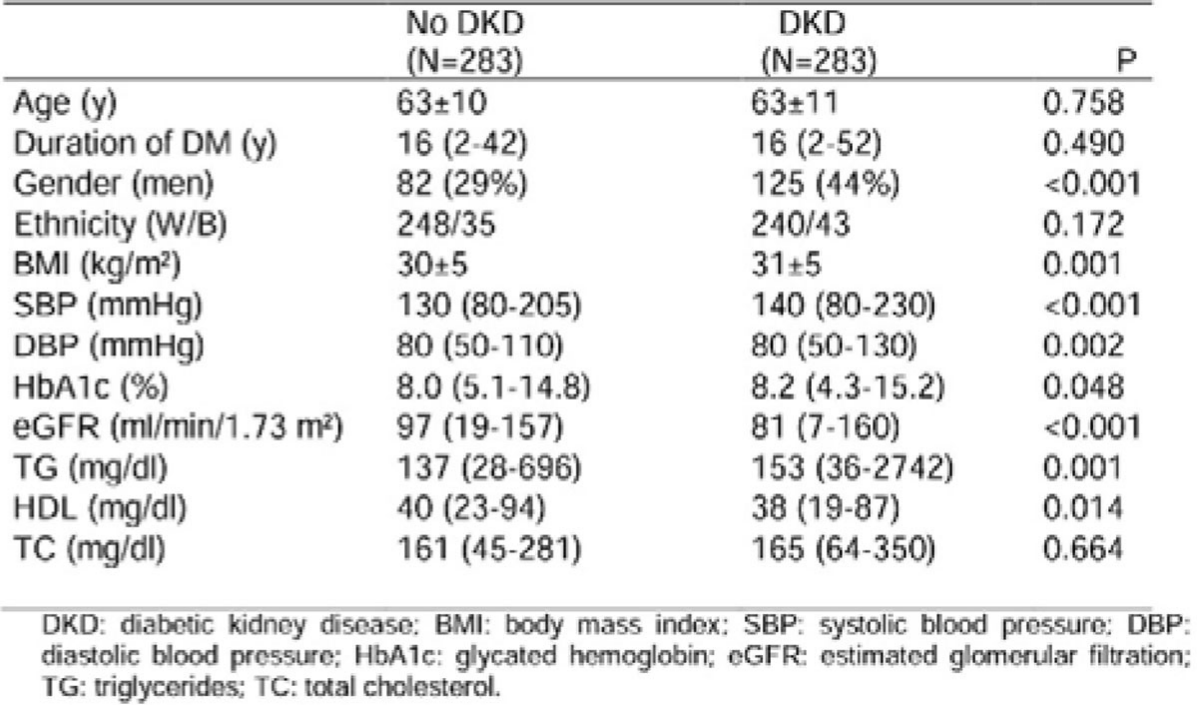
Clinical and laboratory characteristics of DKD patients according to the presence of elevated UAE only.

**Figure 2:**
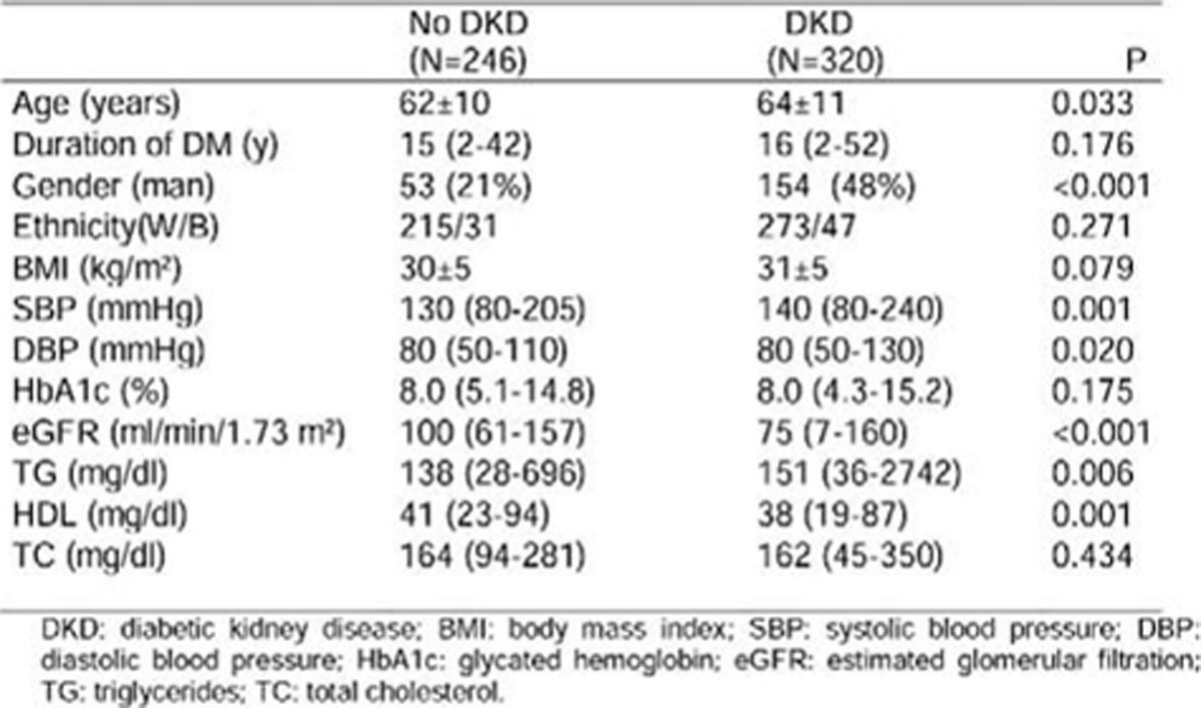
Clinical and laboratory characteristics of DKD patients according to the presence of elevated UAE and/or reduced GFR.

Mean age was 63±11 yrs., 37% men, 86% white, 10% smokers, DM duration 16 yrs. When evaluated by elevated UAE only, 50% of the patients presented DKD. Table 1 shows the profile of these patients (Fig 1). When defined by both UAE and GFR, 57% presented DKD, and Table 2 shows the profile of these patients (Fig 2).

## Conclusion

DKD cases would be missed if only UAE is taken into account.

